# *N*,*N*′-Bis(4-chloro­phenyl­sulfon­yl)­adipamide

**DOI:** 10.1107/S1600536811030029

**Published:** 2011-07-30

**Authors:** Vinola Z. Rodrigues, Sabine Foro, B. Thimme Gowda

**Affiliations:** a Department of Chemistry, Mangalore University, Mangalagangotri 574 199, Mangalore, India; b Institute of Materials Science, Darmstadt University of Technology, Petersenstrasse 23, D-64287 Darmstadt, Germany

## Abstract

In the title compound, C_18_H_18_Cl_2_N_2_O_6_S_2_, the asymmetric unit contains half a mol­ecule with a center of symmetry at the mid-point of the central C—C bond. The dihedral angle between the benzene ring and the SO_2_—NH—C(O) segment in the two halves of the mol­ecule is 83.5 (2)°. In the crystal, N—H⋯O(S) inter­molecular hydrogen bonds link the mol­ecules into infinite chains running along the *c* axis. The O atom involved in the hydrogen bond has a longer S—O bond than the other O atom bonded to S [1.403 (4) *versus* 1.361 (4) Å].

## Related literature

For hydrogen-bonding preferences of sulfonamides, see; Adsmond & Grant (2001 ▶). For our studies on the effects of substituents on the structures of *N*-(ar­yl)-amides, see: Bhat & Gowda (2000 ▶); Gowda *et al.* (2000 ▶, 2007 ▶). For those on *N*-(aryl­sulfon­yl)-amides, see: Rodrigues *et al.* (2011**a* ▶,b* ▶). For those on *N*-(ar­yl)-aryl­sulfonamides, see: Gowda *et al.* (2005 ▶).
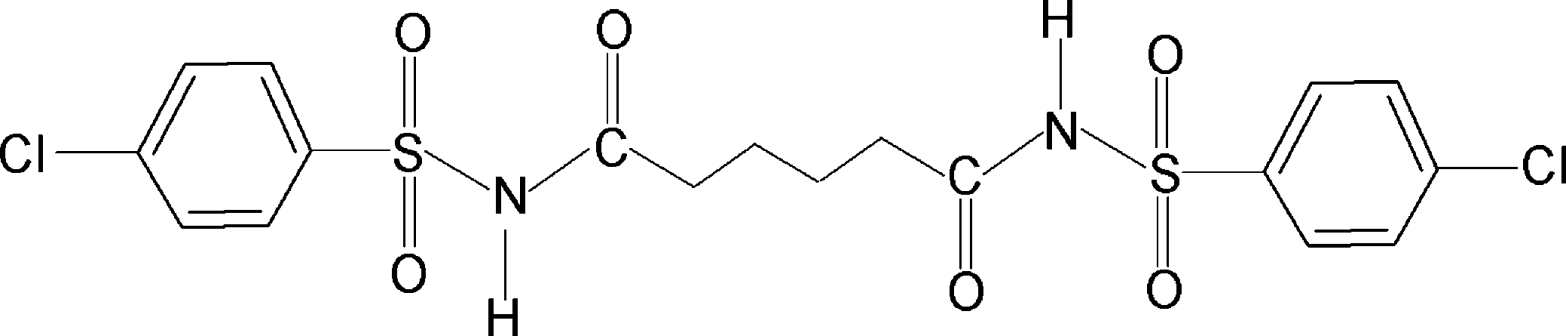


## Experimental

### 

#### Crystal data

C_18_H_18_Cl_2_N_2_O_6_S_2_
*M* *_r_* = 493.36
Triclinic, 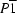

*a* = 5.593 (1) Å
*b* = 8.827 (2) Å
*c* = 9.908 (2) Å
α = 89.28 (2)°
β = 87.75 (2)°
γ = 81.16 (1)°
*V* = 482.96 (17) Å^3^
*Z* = 1
Mo *K*α radiation
μ = 0.60 mm^−1^
*T* = 293 K
0.12 × 0.08 × 0.04 mm

#### Data collection

Oxford Diffraction Xcalibur diffractometer with a Sapphire CCD detector
Absorption correction: multi-scan (*CrysAlis RED*; Oxford Diffraction, 2009 ▶) *T*_min_ = 0.932, *T*_max_ = 0.977
2942 measured reflections
1757 independent reflections
775 reflections with *I* > 2σ(*I*)
*R*_int_ = 0.063

#### Refinement

*R*[*F* ^2^ > 2σ(*F* ^2^)] = 0.081
*wR*(*F* ^2^) = 0.104
*S* = 0.99
1757 reflections
139 parameters
2 restraints
H atoms treated by a mixture of independent and constrained refinement
Δρ_max_ = 0.41 e Å^−3^
Δρ_min_ = −0.36 e Å^−3^

### 

Data collection: *CrysAlis CCD* (Oxford Diffraction, 2009 ▶); cell refinement: *CrysAlis RED* (Oxford Diffraction, 2009 ▶); data reduction: *CrysAlis RED*; program(s) used to solve structure: *SHELXS97* (Sheldrick, 2008 ▶); program(s) used to refine structure: *SHELXL97* (Sheldrick, 2008 ▶); molecular graphics: *PLATON* (Spek, 2009 ▶); software used to prepare material for publication: *SHELXL97*.

## Supplementary Material

Crystal structure: contains datablock(s) I, global. DOI: 10.1107/S1600536811030029/zj2018sup1.cif

Structure factors: contains datablock(s) I. DOI: 10.1107/S1600536811030029/zj2018Isup2.hkl

Supplementary material file. DOI: 10.1107/S1600536811030029/zj2018Isup3.cml

Additional supplementary materials:  crystallographic information; 3D view; checkCIF report

## Figures and Tables

**Table 1:** Hydrogen-bond geometry (Å, °)

*D*—H⋯*A*	*D*—H	H⋯*A*	*D*⋯*A*	*D*—H⋯*A*
N1—H1N⋯O2^i^	0.85 (2)	2.03 (3)	2.839 (7)	160 (6)

Symmetry code: (i) 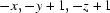.

**Fig. 1.**
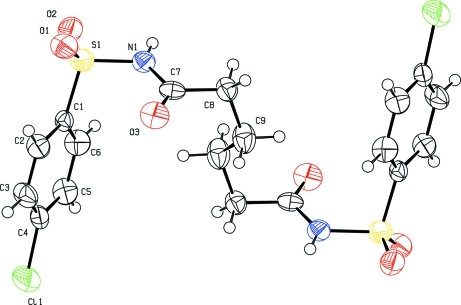
Molecular structure of (I) showing the atom labelling scheme. Displacement ellipsoids are drawn at the 50% probability level and H atoms are represented as small spheres of arbitrary radii.

**Fig. 2.**
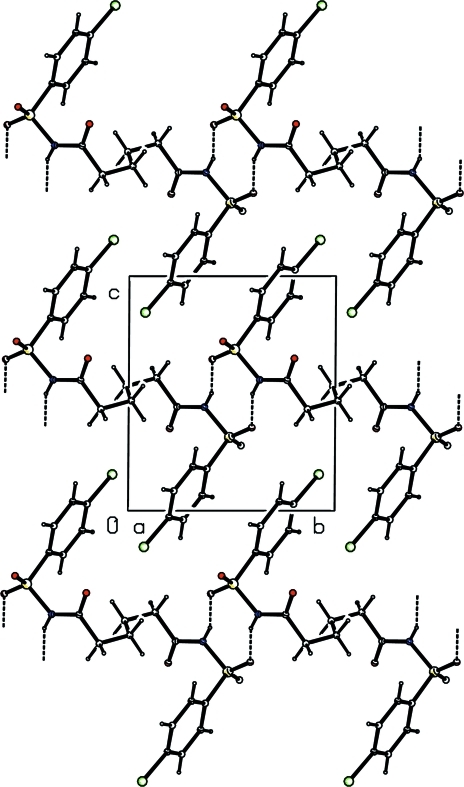
Molecular packing of the title compound with hydrogen bonding shown as dashed lines.

## supplementary crystallographic information

### Comment

The amide moiety is an important constituent of many biologically significant compounds. As part of our studies on the effects of ring and side chain substitutions on the structures of *N*-(aryl)-amides (Bhat & Gowda, 2000; Gowda *et al.*, 2000, 2007), *N*-(arylsulfonyl)-amides (Rodrigues *et al.*, 2011**a*,b*) and *N*-(aryl)-arylsulfonamides (Gowda *et al.*, 2005), the crystal structure of *N*,*N*-bis(4-chlorophenylsulfonyl)- adipamide has been determined (I) (Fig. 1).

In the two C—SO_2_—NH—CO—CH_2_—CH_2_ central segments of the structure, the N—H, C=O and C—H bonds are *anti* to the adjacent bonds, similar to that observed in *N*,*N*- bis(2-chlorophenylsulfonyl)-adipamide (II) (Rodrigues *et al.*, 2011*a*) and *N*,*N*-bis(4-chlorophenylsulfonyl)-suberamide (III) (Rodrigues *et al.*, 2011*b*). The orientations of sulfonamide groups with respect to the attached phenyl rings are given by the torsion angles of C2—C1—S1—N1 = -117.1 (6)° and C6—C1—S1—N1 = 60.5 (6)°. The molecule is bent at the S atom with the C1—S1—N1—C7 torsion angle of 55.0 (6)°, compared to the value of -65.1 (6)° in (II).

The dihedral angle between the benzene ring and the SO_2_—NH—C(O) segment in the two halves of the molecule is 83.5 (2)°, compared to the values of 89.6 (2)° in (II) and 79.5 (2)° in (III).

N—H···O2(S) H-bond formation results in an S=O2 bond longer than the S=O1 bond [1.403 (4)Å *versus* 1.361 (4) Å]. A series of N—H···O(S) intermolecular hydrogen bonds (Table 1) link the molecules into infinite chains running along *c*-axis (Fig. 2). The hydrogen bonding preferences of sulfonamides is described elsewhere (Adsmond & Grant, 2001)

### Experimental

*N*,*N*-Bis(4-chlorophenylsulfonyl)-adipamide was prepared by refluxing a mixture of adipic acid (0.01 mol) with 4-chlorobenzenesulfonamide (0.02 mol) and POCl_3_ for 1 hr on a water bath. The reaction mixture was allowed to cool and added ether to it. The solid product obtained was filtered, washed thoroughly with ether and hot ethanol. The compound was recrystallized to the constant melting point and was characterized by its infrared and NMR spectra.

Needle like colorless single crystals used in the X-ray diffraction studies were grown by a slow evaporation of a solution of the compound in ethanol at room temperature.

### Refinement

The H atom of the NH group was located in a difference map and later restrained to N—H = 0.86 (2) %A. The other H atoms were positioned with idealized geometry using a riding model with the aromatic C—H = 0.93Å and the methylene C—H = 0.97 Å. All H atoms were refined with isotropic displacement parameters (set to 1.2 times of the *U*_eq_ of the parent atom).

The distance C1—C6 in the benzene ring was restrained to 1.39 (1) Å.

### Crystal data

**Table:**

C_18_H_18_Cl_2_N_2_O_6_S_2_	*Z* = 1
*M**_r_* = 493.36	*F*(000) = 254
Triclinic, *P*1	*D*_x_ = 1.696 Mg m^−^^3^
Hall symbol: -P 1	Mo *K*α radiation, λ = 0.71073 Å
*a* = 5.593 (1) Å	Cell parameters from 528 reflections
*b* = 8.827 (2) Å	θ = 3.1–28.0°
*c* = 9.908 (2) Å	µ = 0.60 mm^−^^1^
α = 89.28 (2)°	*T* = 293 K
β = 87.75 (2)°	Needle, colourless
γ = 81.16 (1)°	0.12 × 0.08 × 0.04 mm
*V* = 482.96 (17) Å^3^	

### Data collection

**Table:**

Oxford Diffraction Xcalibur diffractometer with a Sapphire CCD detector	1757 independent reflections
Radiation source: fine-focus sealed tube	775 reflections with *I* > 2σ(*I*)
graphite	*R*_int_ = 0.063
Rotation method data acquisition using ω scans	θ_max_ = 25.4°, θ_min_ = 3.1°
Absorption correction: multi-scan (*CrysAlis RED*; Oxford Diffraction, 2009)	*h* = −6→6
*T*_min_ = 0.932, *T*_max_ = 0.977	*k* = −10→10
2942 measured reflections	*l* = −11→11

### Refinement

**Table:**

Refinement on *F*^2^	Primary atom site location: structure-invariant direct methods
Least-squares matrix: full	Secondary atom site location: difference Fourier map
*R*[*F*^2^ > 2σ(*F*^2^)] = 0.081	Hydrogen site location: inferred from neighbouring sites
*wR*(*F*^2^) = 0.104	H atoms treated by a mixture of independent and constrained refinement
*S* = 0.99	*w* = 1/[σ^2^(*F*_o_^2^) + (0.*P*)^2^] where *P* = (*F*_o_^2^ + 2*F*_c_^2^)/3
1757 reflections	(Δ/σ)_max_ = 0.007
139 parameters	Δρ_max_ = 0.41 e Å^−^^3^
2 restraints	Δρ_min_ = −0.36 e Å^−^^3^

### Special details

**Table:**

Geometry. All e.s.d.'s (except the e.s.d. in the dihedral angle between two l.s. planes) are estimated using the full covariance matrix. The cell e.s.d.'s are taken into account individually in the estimation of e.s.d.'s in distances, angles and torsion angles; correlations between e.s.d.'s in cell parameters are only used when they are defined by crystal symmetry. An approximate (isotropic) treatment of cell e.s.d.'s is used for estimating e.s.d.'s involving l.s. planes.
Refinement. Refinement of *F*^2^ against ALL reflections. The weighted *R*-factor *wR* and goodness of fit *S* are based on *F*^2^, conventional *R*-factors *R* are based on *F*, with *F* set to zero for negative *F*^2^. The threshold expression of *F*^2^ > σ(*F*^2^) is used only for calculating *R*-factors(gt) *etc*. and is not relevant to the choice of reflections for refinement. *R*-factors based on *F*^2^ are statistically about twice as large as those based on *F*, and *R*- factors based on ALL data will be even larger.

### Fractional atomic coordinates and isotropic or equivalent isotropic displacement parameters (Å^2^)

**Table:**

	*x*	*y*	*z*	*U*_iso_*/*U*_eq_	
C1	0.1041 (11)	0.6193 (7)	0.8224 (6)	0.0293 (17)	
C2	0.2221 (12)	0.6416 (7)	0.9348 (6)	0.042 (2)	
H2	0.3810	0.5932	0.9421	0.051*	
C3	0.1175 (13)	0.7325 (8)	1.0388 (7)	0.047 (2)	
H3	0.2030	0.7448	1.1155	0.057*	
C4	−0.1031 (13)	0.8010 (8)	1.0280 (7)	0.042 (2)	
C5	−0.2239 (12)	0.7794 (8)	0.9163 (7)	0.045 (2)	
H5	−0.3822	0.8291	0.9100	0.054*	
C6	−0.1242 (11)	0.6877 (7)	0.8116 (7)	0.0433 (19)	
H6	−0.2120	0.6738	0.7361	0.052*	
C7	0.3253 (12)	0.7659 (8)	0.5567 (7)	0.0357 (18)	
C8	0.2725 (11)	0.8657 (7)	0.4335 (6)	0.0394 (18)	
H8A	0.4234	0.8879	0.3921	0.047*	
H8B	0.1936	0.8111	0.3683	0.047*	
C9	0.1140 (10)	1.0122 (8)	0.4692 (6)	0.054 (2)	
H9A	0.1970	1.0689	0.5309	0.065*	
H9B	0.0846	1.0738	0.3881	0.065*	
N1	0.2328 (10)	0.6336 (6)	0.5582 (5)	0.0371 (15)	
H1N	0.149 (9)	0.600 (6)	0.499 (4)	0.044*	
O1	0.4677 (8)	0.4551 (5)	0.7221 (4)	0.0501 (14)	
O2	0.0961 (8)	0.4035 (5)	0.6456 (4)	0.0508 (14)	
O3	0.4340 (7)	0.8007 (5)	0.6492 (5)	0.0491 (14)	
Cl1	−0.2314 (4)	0.9221 (2)	1.15456 (19)	0.0695 (7)	
S1	0.2378 (4)	0.5111 (2)	0.68742 (19)	0.0419 (5)	

### Atomic displacement parameters (Å^2^)

**Table:**

	*U*^11^	*U*^22^	*U*^33^	*U*^12^	*U*^13^	*U*^23^
C1	0.032 (4)	0.030 (4)	0.026 (4)	−0.004 (4)	−0.006 (3)	0.003 (3)
C2	0.035 (5)	0.049 (5)	0.039 (5)	0.004 (4)	−0.001 (4)	0.002 (4)
C3	0.047 (5)	0.060 (6)	0.031 (5)	0.005 (5)	−0.011 (4)	−0.001 (4)
C4	0.049 (5)	0.042 (5)	0.031 (5)	0.002 (4)	0.004 (4)	0.009 (4)
C5	0.026 (4)	0.056 (5)	0.049 (5)	0.005 (4)	0.000 (4)	0.005 (4)
C6	0.032 (4)	0.050 (5)	0.048 (5)	−0.004 (4)	−0.012 (4)	0.001 (4)
C7	0.024 (4)	0.040 (5)	0.041 (5)	−0.001 (4)	0.004 (4)	−0.013 (4)
C8	0.035 (4)	0.045 (5)	0.036 (5)	0.000 (4)	0.000 (3)	0.005 (4)
C9	0.042 (5)	0.064 (6)	0.053 (5)	0.001 (5)	−0.001 (4)	0.017 (4)
N1	0.041 (4)	0.037 (4)	0.035 (4)	−0.008 (3)	−0.010 (3)	−0.005 (3)
O1	0.036 (3)	0.058 (3)	0.050 (3)	0.013 (3)	−0.006 (2)	−0.003 (3)
O2	0.067 (4)	0.042 (3)	0.047 (3)	−0.017 (3)	−0.018 (3)	0.000 (3)
O3	0.031 (3)	0.062 (4)	0.055 (4)	−0.008 (3)	−0.012 (3)	−0.005 (3)
Cl1	0.0784 (16)	0.0701 (16)	0.0514 (14)	0.0107 (12)	0.0185 (12)	−0.0023 (12)
S1	0.0447 (13)	0.0403 (13)	0.0394 (12)	−0.0009 (11)	−0.0076 (10)	−0.0022 (11)

### Geometric parameters (Å, °)

**Table:**

C1—C6	1.333 (6)	C7—N1	1.348 (7)
C1—C2	1.348 (8)	C7—C8	1.508 (8)
C1—S1	1.731 (6)	C8—C9	1.489 (7)
C2—C3	1.370 (7)	C8—H8A	0.9700
C2—H2	0.9300	C8—H8B	0.9700
C3—C4	1.295 (8)	C9—C9^i^	1.437 (10)
C3—H3	0.9300	C9—H9A	0.9700
C4—C5	1.350 (8)	C9—H9B	0.9700
C4—Cl1	1.719 (7)	N1—S1	1.664 (6)
C5—C6	1.371 (7)	N1—H1N	0.85 (2)
C5—H5	0.9300	O1—S1	1.361 (4)
C6—H6	0.9300	O2—S1	1.403 (4)
C7—O3	1.188 (7)		
			
C6—C1—C2	119.0 (6)	C9—C8—C7	111.2 (5)
C6—C1—S1	117.8 (5)	C9—C8—H8A	109.4
C2—C1—S1	123.2 (5)	C7—C8—H8A	109.4
C1—C2—C3	122.7 (7)	C9—C8—H8B	109.4
C1—C2—H2	118.7	C7—C8—H8B	109.4
C3—C2—H2	118.7	H8A—C8—H8B	108.0
C4—C3—C2	118.6 (7)	C9^i^—C9—C8	112.4 (7)
C4—C3—H3	120.7	C9^i^—C9—H9A	109.1
C2—C3—H3	120.7	C8—C9—H9A	109.1
C3—C4—C5	119.4 (7)	C9^i^—C9—H9B	109.1
C3—C4—Cl1	119.0 (6)	C8—C9—H9B	109.1
C5—C4—Cl1	121.6 (6)	H9A—C9—H9B	107.9
C4—C5—C6	123.1 (7)	C7—N1—S1	125.5 (5)
C4—C5—H5	118.4	C7—N1—H1N	128 (4)
C6—C5—H5	118.4	S1—N1—H1N	106 (4)
C1—C6—C5	117.2 (6)	O1—S1—O2	116.6 (3)
C1—C6—H6	121.4	O1—S1—N1	112.0 (3)
C5—C6—H6	121.4	O2—S1—N1	103.7 (3)
O3—C7—N1	121.0 (7)	O1—S1—C1	106.7 (3)
O3—C7—C8	123.7 (7)	O2—S1—C1	112.4 (3)
N1—C7—C8	115.3 (6)	N1—S1—C1	105.0 (3)
			
C6—C1—C2—C3	−0.3 (10)	C7—C8—C9—C9^i^	−59.2 (9)
S1—C1—C2—C3	177.3 (5)	O3—C7—N1—S1	4.7 (9)
C1—C2—C3—C4	−0.6 (11)	C8—C7—N1—S1	−173.2 (4)
C2—C3—C4—C5	0.8 (11)	C7—N1—S1—O1	−60.3 (6)
C2—C3—C4—Cl1	−177.1 (5)	C7—N1—S1—O2	173.2 (5)
C3—C4—C5—C6	−0.2 (11)	C7—N1—S1—C1	55.0 (6)
Cl1—C4—C5—C6	177.8 (5)	C6—C1—S1—O1	179.5 (5)
C2—C1—C6—C5	1.0 (10)	C2—C1—S1—O1	1.8 (6)
S1—C1—C6—C5	−176.8 (4)	C6—C1—S1—O2	−51.6 (6)
C4—C5—C6—C1	−0.8 (10)	C2—C1—S1—O2	130.8 (5)
O3—C7—C8—C9	−63.7 (9)	C6—C1—S1—N1	60.5 (6)
N1—C7—C8—C9	114.2 (6)	C2—C1—S1—N1	−117.1 (6)

Symmetry codes: (i) −*x*, −*y*+2, −*z*+1.

### Hydrogen-bond geometry (Å, °)

**Table:**

*D*—H···*A*	*D*—H	H···*A*	*D*···*A*	*D*—H···*A*
N1—H1N···O2^ii^	0.85 (2)	2.03 (3)	2.839 (7)	160 (6)

Symmetry codes: (ii) −*x*, −*y*+1, −*z*+1.
